# Supplementary material for: HIVprotI: an integrated web based platform for prediction and design of HIV proteins inhibitors
Source: J Cheminform. 2018 Mar 9;10:12. doi: 10.1186/s13321-018-0266-y (PMC5845081; doi:10.1186/s13321-018-0266-y)
Supplement: Supplementary file 2 — Additional file 2. Source code of HIVProtI web server. [file 13321_2018_266_MOESM2_ESM.zip › HIVprotI_Source-code/hivprotI/weka-3-6-6/doc/weka/classifiers/trees/package-tree.html]

weka.classifiers.trees Class Hierarchy


---


|  |  |  |
| --- | --- | --- |
| **Overview** \| **Package** \| Class \| **Tree** \| **Deprecated** \| **Index** \| **Help** \| **Weka's home** | |  |
| **PREV**   **NEXT** | **FRAMES**    **NO FRAMES**     **All Classes** |  |


---


## Hierarchy For Package weka.classifiers.trees

**Package Hierarchies:**: All Packages

---

## Class Hierarchy

- java.lang.Object
  - weka.classifiers.**Classifier** (implements weka.core.CapabilitiesHandler, java.lang.Cloneable, weka.core.OptionHandler, weka.core.RevisionHandler, java.io.Serializable)
    - weka.classifiers.trees.**ADTree** (implements weka.core.AdditionalMeasureProducer, weka.core.Drawable, weka.classifiers.IterativeClassifier, weka.core.OptionHandler, weka.core.TechnicalInformationHandler, weka.core.WeightedInstancesHandler)- weka.classifiers.trees.**DecisionStump** (implements weka.classifiers.Sourcable, weka.core.WeightedInstancesHandler)- weka.classifiers.trees.**FT** (implements weka.core.AdditionalMeasureProducer, weka.core.Drawable, weka.core.OptionHandler, weka.core.TechnicalInformationHandler)- weka.classifiers.trees.**Id3** (implements weka.classifiers.Sourcable, weka.core.TechnicalInformationHandler)- weka.classifiers.trees.**J48** (implements weka.core.AdditionalMeasureProducer, weka.core.Drawable, weka.core.Matchable, weka.core.OptionHandler, weka.classifiers.Sourcable, weka.core.Summarizable, weka.core.TechnicalInformationHandler, weka.core.WeightedInstancesHandler)- weka.classifiers.trees.**J48graft** (implements weka.core.AdditionalMeasureProducer, weka.core.Drawable, weka.core.Matchable, weka.core.OptionHandler, weka.classifiers.Sourcable, weka.core.Summarizable, weka.core.TechnicalInformationHandler, weka.core.WeightedInstancesHandler)- weka.classifiers.trees.**LADTree** (implements weka.core.AdditionalMeasureProducer, weka.core.Drawable, weka.core.TechnicalInformationHandler)- weka.classifiers.trees.**LMT** (implements weka.core.AdditionalMeasureProducer, weka.core.Drawable, weka.core.OptionHandler, weka.core.TechnicalInformationHandler)- weka.classifiers.trees.m5.**M5Base** (implements weka.core.AdditionalMeasureProducer, weka.core.TechnicalInformationHandler)
                      - weka.classifiers.trees.**M5P** (implements weka.core.Drawable)
                      - weka.classifiers.trees.**NBTree** (implements weka.core.AdditionalMeasureProducer, weka.core.Drawable, weka.core.Summarizable, weka.core.TechnicalInformationHandler, weka.core.WeightedInstancesHandler)- weka.classifiers.trees.**RandomForest** (implements weka.core.AdditionalMeasureProducer, weka.core.OptionHandler, weka.core.Randomizable, weka.core.TechnicalInformationHandler, weka.core.WeightedInstancesHandler)- weka.classifiers.**RandomizableClassifier** (implements weka.core.Randomizable)
                            - weka.classifiers.trees.**BFTree** (implements weka.core.AdditionalMeasureProducer, weka.core.TechnicalInformationHandler)- weka.classifiers.trees.**SimpleCart** (implements weka.core.AdditionalMeasureProducer, weka.core.TechnicalInformationHandler)
                            - weka.classifiers.trees.**RandomTree** (implements weka.core.Drawable, weka.core.OptionHandler, weka.core.Randomizable, weka.core.WeightedInstancesHandler)- weka.classifiers.trees.**REPTree** (implements weka.core.AdditionalMeasureProducer, weka.core.Drawable, weka.core.OptionHandler, weka.classifiers.Sourcable, weka.core.WeightedInstancesHandler)- weka.classifiers.trees.**UserClassifier** (implements weka.core.Drawable, weka.core.TechnicalInformationHandler, weka.gui.treevisualizer.TreeDisplayListener, weka.gui.visualize.VisualizePanelListener)

---


|  |  |  |
| --- | --- | --- |
| **Overview** \| **Package** \| Class \| **Tree** \| **Deprecated** \| **Index** \| **Help** \| **Weka's home** | |  |
| **PREV**   **NEXT** | **FRAMES**    **NO FRAMES**     **All Classes** |  |


---
